# Cell-Based Tracers as Trojan Horses for Image-Guided Surgery

**DOI:** 10.3390/ijms22020755

**Published:** 2021-01-13

**Authors:** Vincent Q. Sier, Margreet R. de Vries, Joost R. van der Vorst, Alexander L. Vahrmeijer, Cornelis van Kooten, Luis J. Cruz, Lioe-Fee de Geus-Oei, Valerie Ferreira, Cornelis F. M. Sier, Frauke Alves, Munitta Muthana

**Affiliations:** 1 Department of Surgery, Leiden University Medical Center, 2300 RC Leiden, The Netherlands; v.q.sier@lumc.nl (V.Q.S.); m.r.de_vries@lumc.nl (M.R.d.V.); j.r.van_der_vorst@lumc.nl (J.R.v.d.V.); a.l.vahrmeijer@lumc.nl (A.L.V.); 2 Department of Nephrology, Leiden University Medical Center, 2300 RC Leiden, The Netherlands; c.van_kooten@lumc.nl; 3 Department of Radiology, Translational Nanomaterials and Imaging Group, Leiden University Medical Center, 2300 RC Leiden, The Netherlands; l.j.cruz_ricondo@lumc.nl; 4 Department of Radiology, Section of Nuclear Medicine, Leiden University Medical Center, 2300 RC Leiden, The Netherlands; l.f.de_geus-oei@lumc.nl; 5 Biomedical Photonic Imaging Group, University of Twente, 7522 NB Enschede, The Netherlands; 6 Department of Research and Development, UniQure, 1100 DA Amsterdam, The Netherlands; v.sier-ferreira@uniqure.com; 7 Percuros B.V. Leiden, 2333 CL Leiden, The Netherlands; 8 Translational Molecular Imaging, Clinic of Hematology and Medical Oncology, Institute of Diagnostic and Interventional Radiology, University Medicine Center Göttingen and Max-Planck-Institute for Experimental Medicine, 37075 Göttingen, Germany; falves@gwdg.de; 9 Department of Infection and Immunity, University of Sheffield, Sheffield S10 2RX, UK; m.muthana@sheffield.ac.uk

**Keywords:** cell-based imaging, near-infrared, nuclear imaging, magnetic resonance imaging, leukocyte, mesenchymal stromal cell, platelets, extracellular vesicle, microorganisms, nanoparticle

## Abstract

Surgeons rely almost completely on their own vision and palpation to recognize affected tissues during surgery. Consequently, they are often unable to distinguish between different cells and tissue types. This makes accurate and complete resection cumbersome. Targeted image-guided surgery (IGS) provides a solution by enabling real-time tissue recognition. Most current targeting agents (tracers) consist of antibodies or peptides equipped with a radiolabel for Positron Emission Tomography (PET) and Single Photon Emission Computed Tomography (SPECT), magnetic resonance imaging (MRI) labels, or a near-infrared fluorescent (NIRF) dye. These tracers are preoperatively administered to patients, home in on targeted cells or tissues, and are visualized in the operating room via dedicated imaging systems. Instead of using these ‘passive’ tracers, there are other, more ‘active’ approaches of probe delivery conceivable by using living cells (macrophages/monocytes, neutrophils, T cells, mesenchymal stromal cells), cell(-derived) fragments (platelets, extracellular vesicles (exosomes)), and microorganisms (bacteria, viruses) or, alternatively, ‘humanized’ nanoparticles. Compared with current tracers, these active contrast agents might be more efficient for the specific targeting of tumors or other pathological tissues (e.g., atherosclerotic plaques). This review provides an overview of the arsenal of possibilities applicable for the concept of cell-based tracers for IGS.

## 1. Introduction to Image-Guided Surgery

Although recent developments in laparoscopic and robotic surgery enable more precise and less destructive operations, these techniques also deprive surgeons from palpation, which is the second-most valuable source of information after visualization. The intraoperative molecular imaging of lesions, which is used in image-guided surgery (IGS), can partially compensate this loss and has already been proven valuable in both oncologic and vascular surgery [1,2]. As an example, during oncologic surgery, IGS might not only help to identify tumor borders, recurrence, and metastases, but also provide more insight in tumor composition, improving the differentiation between healthy and malignant tissue [1]. Analogously, for vascular applications, near-infrared fluorescence (NIRF) is especially promising in the visualization of skin perfusion in patients with severe types of peripheral artery disease [2].

The majority of IGS approaches uses a cell-specific targeting agent equipped with a NIRF dye or radiolabel and are used in conjunction with dedicated imaging systems. The radiotracers may be visualized using Positron Emission Tomography (PET) or Single Positron Emission Computed Tomography (SPECT). In the past years, clinical studies have shown that real-time IGS improves the recognition of tumor tissue significantly, especially in cases of challenging visual inspection and palpation [3,4].

Most of the current IGS tracers are based on monoclonal antibodies, peptides, or aptamers, which are equipped with a NIRF dye (λ emission and λ absorption ranging from 700 to 900 nm). These so-called targeted tracers rely on binding to their tumor-associated target after passive tissue diffusion and are a considerable improvement compared to the first generation of tracers, which consisted basically of non-targeted dyes [5,6]. Positive clinical oncologic IGS results have been obtained with various tracers targeting tumor-associated proteins, including adhesion molecules such as integrins, tyrosine kinase receptors such as vascular endothelial growth factor receptor (VEGFR), epidermal growth factor receptor (EGFR), human epidermal growth factor receptor 2 (HER2), and receptor ligands such as epidermal growth factor (EGF), vascular endothelial growth factor (VEGF), and folate [7,8,9]. However, immunohistochemical evaluation of resected tumors has shown that the malignant cells are not always equally occupied with the tracer. Therefore, distribution throughout the tumor can be heterogeneous, superficial, or patchy [10]. This phenomenon is partly due to the physiological traits of the tracer (e.g., affinity, size, and charge), but it is most likely also related to the characteristics of the lesion. For example, the presence of angiogenic endothelial cells and other stromal cells can strongly influence the diffusion of a tracer through the targeted tissue. An alternative approach to these imaging agents for targeted but otherwise passive strategies would be the use of tracers that are able to actively recognize and penetrate tissues, irrespective of the presence of a single protein on the targeted cells or the presence of less permeable stroma. This is exactly how certain cell types and cell-derived particles naturally behave in response to the presence of specific chemokines excreted by the targeted cells in pathological lesions. Next to cancer, cell-based carriers may be applied in cardiovascular lesions. For instance, preoperative imaging of atherosclerotic plaque activity or instability could be employed to guide the decision of (non-)operative treatments. Furthermore, the progression of (neo)intimal hyperplasia, plaque vulnerability, and vein graft infections could be monitored to schedule timely interventions and prevent further disease progression [11,12,13].

Although holding promise for the treatment and visualization of various diseases, the use of cells for therapy or imaging in clinical applications still must overcome some serious obstacles. Issues considering safety, reproducibility, scalable manufacturing systems, and not in the least the costs will have to be solved. Current developments in cell-based vehicles are frequently based within the field of drug delivery, yet many of the desired characteristics and features overlap with those needed in the field of imaging [14]. In this review, we intend to give a comprehensive overview of possible approaches for the use of cell-based carriers for IGS. The main characteristics determining the choice of future cell-based IGS tracers are compiled in Table 1 and will be discussed accordingly in the various sections. The possibilities of cells as active imaging tracers will be presented in the context of IGS in Section 2, which are accompanied by their associated trafficking mechanisms. A comparable phenomenon by which microorganisms travel to their preferred target tissues and their potential use in IGS will be covered in Section 3. The fourth and last section will concisely focus on nanoparticles, comparing these synthetic alternatives with cell- and microorganism-based carriers. In view of the rapid advances in the field, this review will mainly address the imaging of *tumors*, but wherever appropriate, examples of cell-based imaging strategies of *cardiovascular* diseases will be integrated.

## 2. Cell-Based Carriers for IGS

**Figure 1:**
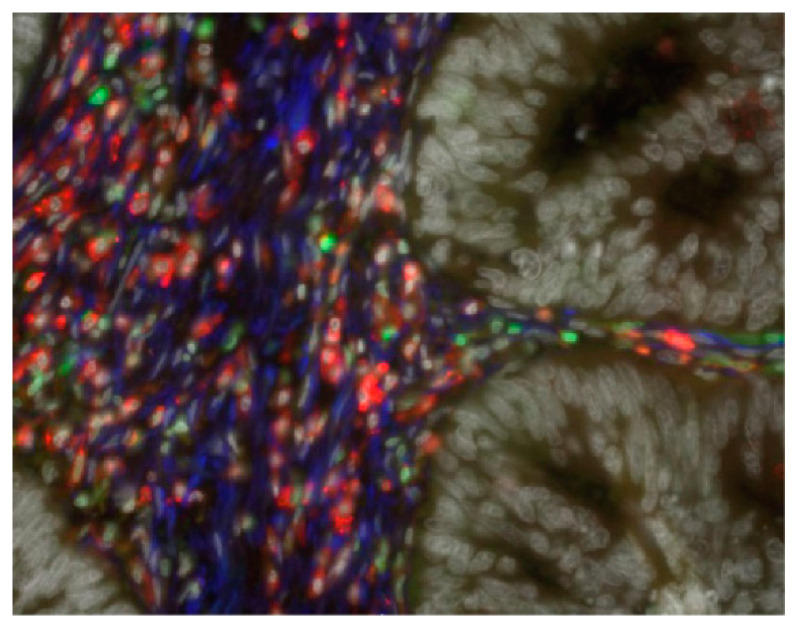
Distribution of cell types within a typical human colon tumor. Colors indicate respectively malignant epithelial cells (gray), immune cells (CD45, red), fibroblasts (fibroblast activation protein, blue), and endothelial cells (CD31, green), as stained in a multiplex analysis using a 40× field. Adapted from Sandberg et al. with permission [25].

Various types of human cells are naturally equipped to migrate actively in the direction of certain parts of the body in response to chemotactic substances such as chemokines, leukotrienes, eicosanoids, and reactants of the complement cascade [15]. Particularly, cells from either the innate immune system (e.g., monocytes/macrophages, neutrophils) or adaptive immune system (e.g., T cells, B cells) are commonly found within wounds, inflammation sites, cardiovascular lesions, and almost any solid tumor, as schematically indicated in Figure 1 [16,17]. In addition, mesenchymal cells (i.e., fibroblasts) and platelets are also commonly found in the activated stroma of most pathological lesions [18,19]. Due to the active recruitment of immune cells to tumor sites, recent studies have evaluated the concept of cells as vehicles for the delivery of therapeutic agents, such as the use of macrophages to target human prostate tumor xenografts in a mouse model [20,21]. Similarly, NIRF- or radiolabeled versions of these cells should be able to reach tumors actively via the circulation and might therefore be more efficient than the commonly used ‘passive’ targeted agents [22,23]. Labeled circulating cells could in principle out-perform targeted proteins and nanoparticles, as they are physiologically more adapted to actively penetrate tissues such as tumors and vascular lesions. The most obvious cells making use of their natural asset of rapid tissue penetration are neutrophils, macrophages/monocytes, T cells, and natural killer (NK) cells, but circulating platelets and mesenchymal stromal cells are also known to migrate toward wound healing, inflammatory, and tumor sites [22,24]. In order to consider any type of cell as a targeted imaging agent, it should be taken into account that retrieval and subsequent ex vivo labeling of these cells should not change the cells’ properties. Therefore, it is important to design and employ strict regulations for the implementation of all cell-based tracers, ensuring (i) precise knowledge of the effects of cell modifications, (ii) prevention of any type of alteration in cell property or biological behavior that might negatively affect patients, and (iii) preservation of the cell’s targeting and imaging capabilities.

### 2.1. Leukocyte-Based IGS Carriers

Circulating leukocytes form an essential component of the immune system and therefore are particularly equipped to reach any site of the body as part of the natural defense. They are commonly classified into two classes, which are based on cellular lineage: *myeloid* (i.e., granulocytes, monocytes, and macrophages) and *lymphoid* (i.e., T cells, B cells, and Natural killer cells).

#### 2.1.1. Monocyte/Macrophage-Based IGS Carriers

**Figure 2:**
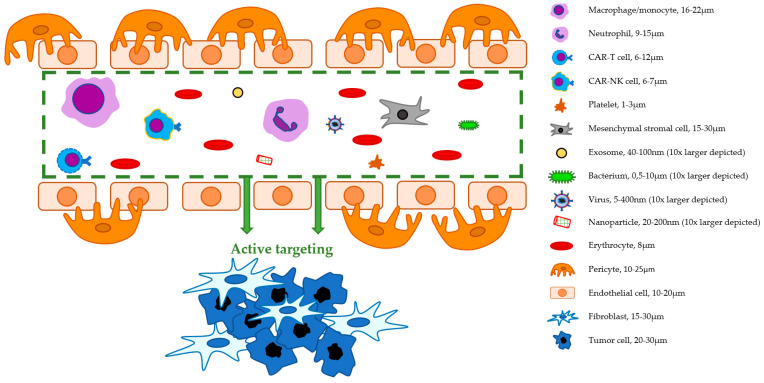
Schematic overview of cell- and nanoparticle-based IGS tracers in a blood vessel near tumor tissue. Sizes of possible IGS tracers differ substantially. Logically, they rely on different mechanisms to reach their target tissue.

Macrophages and their progenitors, monocytes, are important cell types for antigen presentation to lymphocytes and play a key role as effector cells in immune responses. Although they are among the largest in diameter (16–22 μm) (Figure 2 and Table 1), these cells are remarkably flexible and able to penetrate into almost any tissue of the human body. They have strong tumor-homing ability because they are attracted by chronic inflammation, hypoxia, and by tumor cells in response to a gradient of chemo-attractants. An example of a potent chemokine in the attraction of monocytes to tumor sites is monocyte chemoattractant protein-1 (MCP-1, also known as CCL2). This chemokine, produced by malignant tumor cells as well as by stromal cells, contributes to tumor progression [26]. There is an ongoing discussion on the subdivision of macrophages into subtypes with varying characteristics [27]. In general, these cells can be polarized into an inflammatory or tissue repair subtype, with respectively tumor-attacking and tumor-promoting properties. Upon arrival in the tumor microenvironment, they play an active role in various stages of tumor progression: from early carcinogenesis to metastasis [28]. Macrophages also play an important role in cardiovascular diseases and are especially known for their role in atherosclerosis [29].

Macrophages and monocytes could be attractive candidates for IGS, provided that they can be isolated from the circulation, conveniently labeled, and that only the appropriate macrophage type would be used for the desired application. In an example of manipulating macrophage motility, Muthana et al. have demonstrated that magnetically labeled macrophages can be selectively guided to prostate tumors in mice, using a magnetic resonance imaging (MRI) system and pulsed magnetic field gradients [30]. This concept can be extended to the use of different imaging labels such as NIRF- or radiolabeled dyes. In this context, Fu J. et al. have shown that murine macrophage-like cells (RAW264.7 cell line) can be readily labeled with the NIRF dye DiR (KGMP0026) in only 30 min of incubation [31]. Likewise, monocytes isolated from the circulation of patients could be labeled with clinically established NIRF dyes such as indocyanine green (ICG) or IRDye 800CW. Given that macrophages can cross the blood–brain barrier and have already been proposed as targeted drug delivery vehicles [32], one possible application of labeled macrophages as described above would be facilitating the excision of gliomas by IGS. In fact, PET and MRI imaging have been widely combined for visual tracking of tumors and atherosclerotic lesions, using tracers that largely target activated macrophages. For instance, the non-specific probe 18F-Fluorodeoxyglucose (18F-FDG) has been used in oncology for monitoring and staging of cancers and for the assessment of atherosclerotic plaques using PET [33,34].

As indicated, before advocating the implementation of macrophages in IGS, a point of attention is the polarization of macrophage subtypes and their biological role in pathologic lesions. M1 macrophages are associated with infectious and inflammatory diseases, atherosclerosis, and an anti-tumor role, whereas M2 macrophages are associated with an anti-inflammatory role, supporting pro-tumor conditions. Using macrophages for IGS in oncology would require strict control over the type of macrophage used to avoid any unwanted activities that could (i) favor tumor growth, proliferation, and survival and (ii) induce severe side effects. Although a consensus has yet to be reached on what characterizes the terminal stage of macrophage differentiation, it is often thought that macrophages are terminally differentiated and therefore cannot be expanded. Accordingly, the isolation of macrophages from patients for IGS applications would require leukapheresis and elutriation to ensure a sufficient quantity of cells [35].

To summarize, macrophages are attractive candidates as cell-based tracers for IGS in oncology, owing to their tumor-homing capacity, localization in the tumor microenvironment, stealth in blood circulation as monocytes, and to the relative ease with which they can be isolated and labeled with NIRF dyes. In addition, macrophages are abundantly present in cardiovascular lesions and important drivers of atherosclerotic disease. Therefore, they could be targeted in the context of grading and monitoring disease progression. However, macrophage subtypes and possible negative functional changes during cell alterations should be carefully considered. Especially in the context of IGS in oncology, only macrophages with tumor-attacking characteristics should be used. Given these reasons, macrophages should be further explored as tracers for IGS.

#### 2.1.2. Neutrophil-Based IGS Carriers

Neutrophils constitute roughly half of the white blood cells in the body, have a diameter of 9–15 μm, and are the first immune cells to respond to tissue injury or infection sites [36]. Similar to macrophages, neutrophils are actively recruited by chemo- and cytokines that are produced in inflammatory environments, including tumor microenvironments and atherosclerotic plaques [37]. Activated neutrophils use adhesive molecules such as selectins to adhere to vessel walls, after which they transmigrate to reach the site of destination. In this context, Wang et al. discovered that albumin nanoparticles, loaded with the far-red-fluorescent dye Cyanine 5 (Cy5), were internalized by activated, adherent neutrophils in tumor necrosis factor α (TNFα)-challenged mouse cremaster post-capillary venules [38]. Real-time fluorescence showed that 95% of the Cy5-labeled or -conjugated albumin nanoparticles had been internalized by the adherent neutrophils, whereas none of the control particles (albumin-conjugated polystyrene particles or Cy5-conjugated albumin) had been internalized. Furthermore, based on nanoparticle internalization experiments using FcγRIII^−/−^ and wild-type mice, the authors discovered that the FcγRIII receptor contributed to approximately 50% of total albumin nanoparticle internalization by neutrophils [38]. Their findings suggest that in terms of fluorescent imaging, the FcγRIII receptor on activated neutrophils could be exploited for labeling and tracking the neutrophils themselves as well as for delineating the targeted inflammatory site to which these neutrophils ultimately migrate.

Neutrophils have also been harnessed for application in computed tomography (CT) and SPECT imaging. For example, it is feasible to radiolabel autologous neutrophils and to quantify neutrophil accumulation in the lungs of human chronic obstructive pulmonary disease (COPD) patients [39]. Technetium-99m-hexamethylpropyleneamine oxime was used as a radiolabel, and a high rate of reproducibility of the resultant signal among patients at t = 7–10 days was found. Similar to macrophages, neutrophils can cross the blood–brain barrier, enabling them to penetrate brain tissue and target gliomas in mice [40]. Vaas et al. labeled neutrophils with the NIRF tracer LIPO-6S-IDCC, using them for non-toxic intraoperative imaging of brain tumors in mice [41].

In addition to different imaging and labeling applications, neutrophil isolation should be considered. A common misconception is that (labeled) neutrophils generate the majority of the imaging signal, as they are the most common cell type among blood granulocytes. This idea might interfere with precise imaging of the target lesion, as it has been shown that eosinophils in the granulocyte population tend to be radiolabeled with significantly higher efficiency and have a different biodistribution compared to neutrophils [42,43]. The latter is especially important in individuals with inflammatory conditions that involve the upregulation of eosinophils, such as asthma and esophagitis. In this context, the anti-CD16-conjugated magnetic beads approach by Lukawska et al. might be adopted, which allows for the separation of blood eosinophil and neutrophil granulocytes in a human venous blood sample while conserving their migratory phenotype upon intravenous reinfusion [43]. Another issue to consider is that neutrophils are relatively fragile, become easily activated, and die rapidly, which might be a complication for practical use.

Similar to macrophages, neutrophils offer functional advantages that make them attractive tracers for IGS, including their natural abundance in the blood and active homing capabilities. Nonetheless, crucial elements to consider are the accuracy of neutrophil acquisition, the fragility of neutrophils, and the possible alterations of biological properties upon (minimal) stimulation and labeling.

#### 2.1.3. T Cell-Based IGS Carriers

T lymphocytes or T cells (diameter = 6–12 μm; Figure 2 and Table 1) are specialized in the tracking and killing of cells that express foreign peptides. As critical components of adaptive immunity, T cells perform specialized functions such as the tracking or killing of foreign cells, activation of immunological memory against previously confronted antigens, and the suppression of immune responses to prevent autoimmunity. The vigorous infiltration of certain T cells in tumor lesions has been correlated to a positive prognosis in several cancer types, including bladder cancer, metastatic melanoma, ovarian cancer, and renal cell carcinoma [44,45]. In a meta-analysis on the presence of CD3+, CD4+ (helper or “Th”), CD8+ (cytotoxic), or FoxP3+ (regulatory) T cells in solid tumors, Gooden et al. concluded that a high ratio of CD8+ to FoxP3+ T cells, and the presence of both CD3+ and CD8+, each correspond to higher survival rates [46]. High numbers of especially CD8+ T cells are an indication of beneficial immunogenicity, which can result in an improved response to immunotherapies and a more favorable prognosis [45]. Additionally, the presence of Th1 cells, a subtype of CD4+ T cells, is a positive prognostic factor for cancer patients [47].

T cells are currently being explored for targeted tumor imaging and therapy [48]. Chimeric antigen receptor (CAR)-T cells are especially interesting. CAR-T cells are T cells that are genetically modified to express a recombinant antigen recognition domain to optimize the targeting of a patient’s tumor cells. These CAR-T cells are manipulated and expanded ex vivo and subsequently administered to patients. CAR-T cells can be prepared in various ways, either of which is biocompatible; The currently FDA-approved CAR-T cell therapies are based on *autologous* CAR-T cells, in which the original (unmodified) T cells originate from the patient. However, there are increasing efforts at developing *allogenic* CAR-T cells, which are manufactured in large batches and may be used in multiple patients. The major drawback of autologous CAR-T cells is that the acquisition and manipulation of these cells is both time-consuming and expensive. Additionally, problems may arise due to off-target responses such as cytokine release syndrome (treatable with tocilizumab [49]) and neurotoxicity [50]. Moreover, there is a paucity of validated standardized tools for qualifying the potency of CAR-T cells [51].

Interestingly, cytotoxic T cells (also known as cytotoxic T lymphocytes (CTLs)) can be functionalized with nanoparticles for applications in tumor imaging and therapy. For instance, Jones and colleagues prepared nanocapsule-CTL conjugates and tested them as agents for the targeted delivery of immunotherapy to an anatomic site of viral replication in HIV-infected humanized mice [52]. Intriguingly, drug release from these nanocapsules is triggered by specific stimulation of the T cell receptor. By extension, one could merge the tumor-homing properties of CAR-T cells with NIRF dye-labeled or radiolabeled nanoparticles to generate a hybrid nanoparticle/CAR-T tracer. Although we have mostly focused on the imaging of tumors and the tumor microenvironment, there are characteristics of T cells that make them potentially useful for other IGS applications in oncology. For example, tumor-egressing T cells have been shown, in a mouse model, to disseminate to distant tumors and draining lymph nodes, mediating anti-tumor responses at sites of metastasis; Accordingly, a tracer such as the NIRF dye- or radiolabeled nanoparticle/CAR-T hybrid described above might be useful for imaging lymph nodes and distal metastases [53].

On the whole, (CAR-)T cells offer numerous characteristics that suggest their utility for IGS. The combination of their specificity and biocompatibility has great potential, provided that progress will be made regarding the efficiency and costs of T cell acquisition. For successful clinical translation, it is essential that cell-based issues such as unintended altered functions and off-target responses will be controlled or avoided.

#### 2.1.4. NK Cells-Based IGS Carriers

Natural killer (NK) cells, which are subsets of lymphocytes, play an important role in the innate immune system by killing target cells without the need of immunization or prior sensitization. They have a relatively small diameter (6–7 μm) in comparison to other immune cells (Figure 2 and Table 1). To control their cytotoxicity in healthy tissue, inhibitory receptors on the surface of NK cells constrain their activation upon recognition of major histocompatibility complex (MHC) class-I receptors on healthy, nucleus-containing cells. When MHC I expression is absent or abnormal, which commonly occurs in infected cells or cancer cells, the inhibitory signals are lost, and the target cell is killed via a system of lytic granules containing granzymes and perforin proteins [54,55]. In addition to this function, NK cells are known to be involved in modulating both innate and adaptive immune responses through the production of cytokines such as TNFα, interferon γ, macrophage inflammatory protein (MIP)-1α, and MIP-1β [56].

Recently, CAR-NK cells have been described as potent tumor-targeting cells in the context of both direct NK cell-mediated cytotoxicity and the indirect use of NK cells as anti-tumor drug carriers [57,58]. Similar to the concept of the previously described CAR-T cells, CARs are added to the cell membrane of NK cells, improving their tumor-homing abilities. Despite their similarity, these CAR-NK cells have some important advantages compared to CAR-T cells. Firstly, due to their shorter circulation lifetime and less aggressive cytokine release spectra, CAR-NK cells are less associated with harmful side effects such as neurotoxicity and cytokine release syndrome [58,59]. Moreover, to prevent unwanted in vivo proliferation and persistence, NK cells may be irradiated prior to their clinical application [60,61]. Secondly, the use of allogeneic NK cells is less associated with graft-versus-host-disease, allowing for the use of unlimited allogeneic sources such as induced pluripotent stem cells, human embryonic stem cells, umbilical cord blood, peripheral blood mononuclear cells, and NK-92 cells [62,63,64]. The latter is an immortal cell line, originating from an NK cell lymphoma patient, that is easily modified, expanded, and stored under conditions of good manufacturing practice. Thirdly, it is estimated that, whereas the preparation and administration of CAR-T cells in cancer immunotherapy may cost up to 250,000 dollars, engineered NK-92 infusion cycles commonly cost less than 20,000 dollars [65].

Next to the apparent potential of CAR-NK cells for IGS, several limitations should be considered. The largest challenge of CAR-NK cells is the genetic transfection method for engineering NK cells to express CAR receptors. The predominant techniques use lenti- or retroviral gene transduction, but these methods are respectively lacking in terms of efficiency and the possibility of inducing insertion mutations [66,67]. However, it should be noted that developments in this field will in all probability lead to the standardization of more efficient gene transduction methods in the near future [68]. Since the research field of CAR-NK cells is relatively novel as compared to CAR-T cells, most of the current CARs are specifically designed for the latter cell type. Applying these CARs in the context of NK cells might result in general inefficiency and negative alterations in cell properties. Specific CAR-NK cell constructs should be adopted for IGS purposes, such as in the approach of Li et al., who established CAR-NK cells to target melanomas [63].

In the context of IGS, it is feasible to construct aptamer-engineered hepatocellular carcinoma‑specific NK cells that are labeled with ICG and allow for effective imaging in mice [69]. These aptamers, single-stranded oligonucleotides, are designed to specifically recognize and bind their targets. As described by Yang et al., the generation of aptamer-NK cells is straightforward, has a high yield, does not alter cellular properties, and can be carried out within 30 min [70]. MRI imaging of NK cells in vivo has mainly been achieved with the use of superparamagnetic iron-oxide nanoparticles. For instance, the visualization of NK-92 cell lines with superparamagnetic iron-oxide ferucarbotran and ferumoxide was studied by Daldrup-Link et al., showing that genetic modification steps enabled CAR-specific targeting of tumor-associated HER2/neu positive mammary tumors in mice [71]. A more recent study confirmed the clinical feasibility of this technique by labeling murine NK cells with a nanocomplex consisting of heparin, protamine, and ferumoxytol (HPF) to visualize NK cell distribution in a rat model of hepatocellular carcinoma [72]. The biodistribution of both autologous and allogenic in vitro cultured NK cells, radiolabeled with indium-111 oxine, has been investigated in patients with renal cell carcinoma and liver metastases respectively and showed the feasibility of SPECT imaging [73,74]. Next to this, an in vivo antibody-mediated approach was reported by Galli et al., which might overcome issues of indium-111 oxine regarding NK cell viability [75]. They radiolabeled an anti-CD56 monoclonal antibody with technetium-99m that subsequently targeted and imaged NK cells in SCID mice that bore human anaplastic thyroid cancer cells. Meier and colleagues showed that labeling of CAR-NK-92 cells with the PET tracer 18F-FDG allowed for the imaging of implanted HER2/neu-positive tumors in mice at 60 minutes after injection [76].

In summary, (CAR-)NK cells demonstrate promising characteristics as carriers for IGS. CAR-NK cells show less toxicity and graft-versus-host-disease than their CAR-T cell counterparts, are relatively inexpensive to generate, and can be engineered to be highly specific for target tissue. Necessary for the translation of CAR-NK cells to the clinic are advancements in the transduction of CAR genes to increase efficiency and avoid genotoxicity. Moreover, the development of specific CAR constructs for the purpose of CAR-NK cell-based IGS should be encouraged.

### 2.2. Platelet-Based IGS Carriers

Platelets, also known as thrombocytes, are nucleus-free living cell fragments that are primarily present in blood vessels. With an estimated diameter of 1–3 μm (Figure 2 and Table 1), they are smaller than any of the immune cells presented above, facilitating tissue penetration. A human adult can produce up to 5 × 10^11^ platelets a day and maintains a concentration of (150–400) × 10^3^ per μL blood, in contrast to about (4–10) × 10^3^ leukocytes per μL blood [77,78,79]. Platelets are mostly known for targeting vascular lesions, but they also bind to tumor-associated proteins such as fibrinogen, collagens, and integrins. They commonly accumulate in the tumor microenvironment in a process called extravasated platelet aggregation, especially at sites of neoangiogenesis and invasive borders [80,81,82,83]. Platelets are also found on invading and circulating tumor cells, where they play a role in the stimulation of the epithelial-to-mesenchymal transition status of these cells and are associated with chemoresistance [83]. Moreover, platelets can facilitate cancer progression by secreting angiogenesis-inducing bioactive lipids and proteases upon activation by tumor cells [84]. In the context of platelet transfusion for donor-dependent thrombocytopenic patients, the ex vivo storage and generation of thrombocytes has been extensively studied. Prepared platelets that have been transfused within 24–48 h exhibit good survival rates, recovery, and function [85]. The addition of platelet additive solutions, the use of technologies that remove pathogens, and advances in automation have led to further extension of possible storage times. However, this gain in storage time was at the cost of a rapid decrease in quality and nonviability of platelets after five to seven days [85,86]. Platelets can be produced using stem cells of diverse origins by generating mature megakaryocytes ex vivo and subsequently injecting them into experimental subjects based on the process of platelet production in pulmonary capillary beds [77,87]. However, in either case, megakaryocyte maturation and megakaryocyte platelet release still need to be optimized, especially considering the cost of current techniques [87].

Among the main advantages of platelets as candidates for IGS tracers is that they have privileged access to the deepest parts of the body. Platelets gain access to pathologic microenvironments through passive diffusion across pores in surrounding vessels. For example, platelets have demonstrated potential as delivery systems to penetrate the blood–brain barrier for the treatment of malignant glioblastomas and ischemic strokes [88]. The former, being the most common and aggressive form of primary brain tumors, is initially treated with surgery [89]. Intraoperative imaging, using modalities such as MRI, PET, and CT, is necessary to reach maximal resection of the primary tumor [90,91]. It is evident that a combination of radiolabeled platelets in combination with PET-CT or SPECT-CT could improve upon existing imaging techniques for staining tumor tissue and thus ameliorate the accuracy of surgical treatment. In addition, the in vivo lifetime of platelets is perfectly suited for imaging, with a survival time of seven to ten days and a circulation time up to nine days in humans, compared to only about 3 to 9 h of circulation time of nanoparticles [78,92]. This is important, since a longer survival time increases the chance of encountering tumor cells. Moreover, given their size of about 2 μm, which is larger than most synthetic nanoparticles (Figure 2), they are less likely to be purged by scavenging macrophages and liver cells. Together, these factors should allow for excellent penetration at the target site and, consequently, suggest that platelets are strong candidates for imaging purposes. In fact, there is a history in such applications.

Fluorescently labeled platelets have been introduced by Horne in 1975, followed by multiple other groups with studies on similar systems, in which various fluorophores have been coupled to the membranes of platelets [92,93,94]. In the context of a dye-carrying approach, platelets may release their dye in a very controlled manner upon reaching their target, due to the earlier mentioned cancer cell-induced platelet activation. Interestingly, this mechanism can be exploited by manually inducing a release of platelet contents, as demonstrated by Sarkar et al., potentially leading to strong local effects [93]. Similarly, platelets could be engineered to carry NIRF dyes or radioactive tracers and specifically release those at target sites. In addition, platelets can also be marked with radiolabels at ligand‑induced binding sites—epitopes that bind to single-chain antibodies. For instance, a single-chain antibody, targeting the activated integrin αIIbβ3 on platelets and labeled with Indium-111, was used in an in vivo carotid artery thrombosis mouse model to detect wall-adherent activated platelets by using SPECT-CT [95]. Furthermore, another clinically attractive feature of platelets is that they can be quickly adapted for imaging; Repurposing freshly acquired platelets into usable imaging vehicles can typically be completed within 60 minutes, especially when automated [92]. Finally, from a clinical point of view, the imaging of activated platelets could be used in the monitoring of plaque stability within the fields of carotid and heart surgery.

Despite the aforementioned advantages of platelets, there are some disadvantages to consider. Platelets are known to aggregate easily in response to several chemical and physical conditions required for their repurposing [96,97]. One solution to this problem is to incubate the platelets with kabiramide, which is a potent inhibitor of actin polymerization that prevents aggregation [92]. Another point of concern is that the tumor cell-mediated activation of platelets may lead to platelet–tumor cell aggregates, which would then shield the tumor cells from the immune system and facilitate metastatic spread [98]. Additionally, thrombocytes have been shown to induce (primary) tumor growth, cell invasion, and epithelial-to-mesenchymal transition, and they are able to promote angiogenesis and tumor cell settlement at distant sites [98,99]. However, although platelets can facilitate cancer in these ways, the number of platelets used for imaging would be negligible in contrast to the total number that is already circulating, especially considering their circulation lifetime.

Conclusively, human platelets seem appealing candidates for imaging applications. Considering the short time required to repurpose them into imaging vehicles, their widespread availability in the circulation, and their tissue-penetrative capabilities, platelets could serve as excellent cell-based tracers for IGS. Nevertheless, in order to facilitate a translation to the clinic, further research is needed to elucidate the extent and effects of platelet–tumor cell interactions, ensure patient safety, and optimize and standardize production processes. Moreover, an important first step should be to assess the different concepts of platelet-based imaging: (i) labeled platelets accumulating in target tissue and (ii) platelets releasing specific dyes at targeted sites.

### 2.3. Mesenchymal Stromal Cell-Based IGS Carriers

Mesenchymal stromal cells (MSCs), which have already shown therapeutic promise in inflammatory and degenerative diseases, have recently emerged as cell-based (drug) carriers [100,101]. MSCs are multipotent cells that exhibit fibroblast-like morphology and can differentiate into adipocytic, myogenic, osteogenic, and chondrogenic lineages [101,102]. The minimal criteria for the definition of human MSCs, as defined by the International Society for Cell therapy, are very broad, leaving room for different interpretations and, consequently, heterogeneous reports of cell types [100,103]. Nevertheless, MSCs have several traits that make them well suited as carriers for IGS. Importantly, as cells that regulate immune responses, MSCs have inflammation- and tumor-homing capacities, allowing them to migrate to primary tumors, metastases, and vasculature, following inflammatory cues [104,105,106]. This functionality can be of great benefit in cancer patients treated with neoadjuvant radiotherapy, since radiotherapy induces cell injury and inflammation, making MSCs more prone to migrate to the irradiated tumor site.

The side effects and long-term biodistribution of human MSC engraftment have been studied in SCID and non-obese diabetic mice by Francois et al. [107]. Although the authors did not report any side effects in this study, their group had previously demonstrated that human MSCs could be used to help recover irradiated tissues in mice [108]. There exists some evidence that the latter also works in humans with irradiation-induced colitis [109,110]. Additionally, MSCs offer other benefits such as hypo-immunogenicity, amenability to autologous transplantation, relative ease of acquisition, and rapid ex vivo expansion [111].

Furthermore, in the context of imaging, MSCs can be labeled using either or both a radiotracer and an MR contrast agent. As an example, MSCs co-labeled with indium-111 oxine, a clinically commonly used radioactive tracer, and ferumoxides–poly-l-lysine, a combination of a superparamagnetic iron oxide nanoparticle and a DNA transfection agent, allowed for in vivo biodistribution tracking, combining SPECT-CT and MRI in a myocardial infarction mouse model [112]. Regarding NIRF imaging, Kim et al. labeled human bone marrow-derived MSCs with fluorescent nanoparticles and subsequently injected them systemically into glioma-bearing mice [113]. Their results indicate that tracking using NIRF-MSCs is a feasible approach for targeting malignant gliomas, but also that these MSCs become trapped in the lungs, liver, and spleen. One explanation for this phenomenon is a pulmonary first-pass effect due to mechanical entrapment, owing to the relatively large size of MSCs compared to other cells, in particular after ex vivo culture, with diameters ranging from 15 to 30 μm (Figure 2 and Table 1) [101]. This entrapment occurs in vessels of small diameter, explaining the accumulation in the capillary beds of the lungs [114]. Researchers have hypothesized that the redistribution of MSCs in liver and spleen can be ascribed to clearance of apoptotic MSCs by phagocytic monocytes [113,114,115]. Interestingly, the immunosuppressive capacity of MSCs has been linked to MSC apoptosis and subsequent clearance by myeloid cells [115]. Regarding entrapment, not only the large cell volume plays a role, but vascular cell adhesion molecule-1 (VCAM-1) on the cell membrane of endothelial cells seems to be of importance in adhesion to the pulmonary vasculature. Accordingly, inactivation of the VCAM-1 counter-ligand on the surface of MSCs leads to an increased rate of these cells reaching the arterial circulation [114]. In addition to this, MSCs might induce coagulation as a result of the expression of functionally active tissue factor [116]. Although this effect must be taken into consideration, it can easily be reversed through the administration of antithrombin therapy, as reported by Gleeson et al. [116].

All things considered, MSCs have various assets to be a potent vehicle for IGS, especially regarding acquisition and expansion. However, entrapment of MSCs and their coagulation-inducing properties, in addition to the earlier mentioned cell-modification-related reactions, should all be taken into account before strategies toward clinical applications can be developed. The efficiency of target tissue imaging on the one hand and harmful side effects on the other hand, even when treatable, must be carefully weighed against each other.

## 3. Non-Cellular and Bacterial Agents for IGS

### 3.1. Extracellular Vesicle/Exosome-Based IGS Carriers

Extracellular vesicles (EVs) are membrane-enclosed vesicles secreted by cells. They range from 40 nm to 5 μm in diameter, and their main function is to facilitate intercellular communication by transferring RNA, DNA, and proteins between cells. EVs can be divided into three subgroups: (i) exosomes, which are particles with a lipid bilayer that are released from cells and are found in most fluids of the body (diameter: 40–100 nm; Figure 2 and Table 1), (ii) microvesicles (MV), also known as shedding vesicles and ectosomes (diameter: 50 nm–1 μm), which are formed through outward budding and membrane shedding, especially from transformed or injured cells, and (iii) apoptotic bodies (diameter: 50 nm–5 μm), which are parts of the cellular content of apoptotic cells, containing DNA, RNA, and histone proteins [117,118,119]. Given their natural functionality of cell-to-cell transfer of biomolecules, EVs have been modified for their use as drug delivery vehicles: for example, for the administration of anti-cancer agents in mice [120,121]. With respect to imaging applications, the focus of current research lies primarily on exosomes. Therefore, the following paragraphs will cover this topic.

In general, EVs—and exosomes in particular—have many features that make them ideal candidates for IGS tracers. As mentioned above, they are natural carriers of signal molecules (e.g., DNA, miRNAs, siRNAs, lncRNAs, proteins, and lipids) for cell-cell interactions [122]. Moreover, EVs offer good biocompatibility and high physiochemical stability, permeate well through biological barriers (e.g., the blood–brain barrier), and, when used autologously, are non-immunogenic [122,123]. Despite these features, unaltered exosomes will, similar to most nanocarriers, accumulate in the liver, spleen, and kidney, after administration. To address this problem, researchers have functionalized the surface of exosomes with peptides or antibodies that target tumors. Examples of the latter are the GE11- and EGF-positive exosomes, which Ohno et al. created for targeted miRNA delivery to EGFR-expressing breast cancer cells in mice [124]. The exosomes were derived from human embryonic kidney 293 cells that were transfected with a mammalian expression vector encoding GE11 or EGFR [124]. After systemic injection, the exosomes were tracked in vivo using the lipophilic NIRF dye XenoLight DiR. However, despite their tumor-targeting approach, the exosomes still accumulated in the liver 24 h after injection. Nevertheless, long circulation times are not needed for intraoperative tumor imaging: a period from several hours up to three days would suffice. One could even argue that relatively fast systemic clearance is beneficial for the patient, as this would both lead to an improved tissue-to-background ratio and a reduction of possible side effects. Importantly, Ohno et al. did not find any signs of major organ damage, which they verified by hematoxylin and eosin staining of the liver, spleen, and kidney tissue from mice that had received the exosomes [124]. In the context of vascular applications, Wu et al. engineered exomes, derived from inflammatory subtype macrophages, to target, image, and treat atherosclerotic lesions in apolipoprotein E-deficient (ApoE^−/−^) mice on a high-cholesterol diet [125]. Interestingly, these exosomes showed the same inflammation-tropism characteristics of their source cells, which was attributed to surface-bonded chemokine receptors. Moreover, the researchers showed that the electroporation of these exosomes with hexyl 5-aminolevulinate hydrochloride could be exploited to biosynthesize heme, which generated both anti-inflammatory products and the red fluorescent intermediate protoporphyrin IX [125].

The most important characteristic to consider when choosing specific exosomes for any clinical application is that the cellular lineage of a given exosome dictates its composition and behavior. For example, exosomes derived from dendritic cells (DCs), B cells, mast cells, or intestinal epithelial cells all express high levels of functional MHC class-I and class-II molecules, which may elicit unwanted, MHC class-I- and class-II-restricted immune responses in patients treated with non-autologous particles [126]. In the search of an ideal exosome source, Tian et al. utilized exosomes derived from immature murine DCs (imDCs) to deliver chemotherapy in murine models of cancer [127]. Specifically, they engineered imDCs to express lysosome-associated membrane glycoprotein 2b (LAMP2b), loaded them with doxorubicin, and then administered them intravenously to human breast tumor-bearing BALB/c mice. The authors highlighted two critical factors behind their decision to adopt DC-derived membrane vesicles: (i) safety, which has previously been proven in clinical trials and (ii) the surface expression of CD9, which improves cellular drug delivery by enabling direct membrane fusion with target cells [127].

Currently, a major hurdle for exosome usage is that there exists no consensus on preferred purification techniques to isolate and obtain high yields of pure exosomes [128,129]. So far, the most commonly used method is ultracentrifugation. Typically, exosome yields are less than 1 μg exosomal protein per 1 mL of culture medium [130]. In contrast, in most mouse studies, the useful dose of exosomes ranges from 10 to 100 μg exosomal protein per animal [130]. Therefore, the large-scale production of exosomes might be expensive [118]. However, it should be kept in mind that recent developments in the field of exosome isolation, such as size exclusion chromatography, may significantly improve scalability and costs in the near future [131].

To conclude, exosomes are promising targeting agents due to their natural role as carriers of signaling molecules. Moreover, given their natural structural diversity based on cell lineage, as well as the facility with which they can be modified at their surfaces with targeting moieties, exosomes are amenable to custom imaging applications for tumors and vascular diseases. Nonetheless, in order to translate the promising imaging capabilities of exosomes into the clinic, developments in the field of isolation and purification are crucial.

### 3.2. Bacterium-Based IGS Carriers

Recent scientific developments have introduced the use of bacteria for cancer therapy [132,133]. On average, bacterium sizes range from 0.5 to 10.0 μm (Figure 2 and Table 1), enabling them to effectively infiltrate the human body and elicit inflammatory responses. Multiple species of anaerobic bacteria are known to colonize and replicate in hypoxic and necrotic regions of tumors, including species from the *Salmonella*, *Bifidobacterium*, and *Clostridium* genera [134,135]. Bacteria can be easily genetically manipulated in order to express (non-)human and fluorescent proteins of choice. In an ideal situation, one could envision a wide gamut of bacteria functioning as IGS tracers, in which context the species would be chosen according to its specificity for a type of tumor or cardiovascular lesion. 

Intriguingly, whole bacteria have been used in tumor immunotherapy; However, they can be pathogenic and cause adverse reactions in healthy tissue. Thus, any bacterium used as a carrier for imaging agents would have to be completely non-pathogenic and non-toxic. One way to ensure this would be to use naturally non-pathogenic strains such as those found in common food products. Along these lines, Miyaguchi and colleagues reported that the direct injection of *Lactobacillus casei*, which is found in the human gut as well as in many fermented foods, into murine models of cancer provoked a reduction in tumor growth relative to untreated controls [136]. Importantly, they did not find the bacterium in any healthy tissue. In humans, a successful example of the use of mycobacteria in cancer is the bacillus Calmette–Guérin (BCG) therapy used for bladder cancer. BCG therapy has been used as a treatment for non-muscle-invasive bladder cancer for more than 30 years. BCG therapy is highly effective in early stage bladder cancer and has a preventative effect on postoperative recurrence [137]. It is thought that the anti-tumor activities of BCG are caused by activation of the immune system and subsequent inducement of inflammatory responses. However, this anti-tumor effect is also associated with genitourinary side effects such as chemical cystitis (35%), bacterial cystitis (23.3%), macroscopic hematuria (22.6%), and with systemic complications such as persistent high-grade fever (2.9%) [138]. These undesired effects presumably stem from the body’s inflammatory response to BCG and are most likely induced by the process of inflammation. Therefore, inflammation is seen as a double-edged sword in the field of bacterium-based therapies or carriers, entailing an important pathogenic mechanism as well as generating a potent anti-tumor effect.

The therapeutic use of bacteria, even in the case of carefully selected attenuated strains, generally involves risk of adverse events. This is especially true with systemic administration of bacteria, which can provoke hyperimmune reactions (e.g., cytokine storm) with severe consequences for patients, including death [135]. Logically, before any patient is treated with bacteria, caregivers must ensure that species-specific antibiotics are available for immediate use upon any sign of harmful (immune) responses.

An advantage of using bacteria for therapeutics as well as imaging is that bacterial culture systems have been used for decades, which translates to existing infrastructure, well-established manufacturing processes, and low costs [135]. There have been numerous studies on bacteria-based imaging. For instance, Berlec et al. established fluorescence in bacteria by expressing the infrared fluorescent protein IRFP713 in three bacterial species (*Lactobacillus plantarum*, *Lactococcus lactis*, and *Escherichia coli*), subsequently administered the bacteria to mice, and then monitored the live bacteria in the intestinal tracts of the animals [139]. Expression of an additional protein, IRFP682, enabled concomitant near-infrared detection of two different bacterial populations within a single mouse via spectral unmixing. As with bacterial therapeutics, bacterial imaging agents present risks of adverse immune responses in patients. For example, the host immune response can clear the bacteria from the bloodstream, precluding them from colonizing the tumor site at detectable levels [140]. In addition to the selective removal of virulence factors and use of antibiotics, a solution to hyperimmune reactions could lie in genetically manipulating bacteria in order to be replication deficient upon the absence of specific nutrients. A noteworthy example is the A1-R *Salmonella* strain, which is known for its anti-tumor effects, accumulation at tumor sites, and which is auxotrophic for arginine and leucine [141]. Potentially, auxotrophy could be employed to locally stimulate bacteria to replicate, which would increase the tissue-to-background ratio and simultaneously decrease the number of bacteria that need to be injected. Regarding radiolabeling of bacteria, multiple species have been traced in vivo, including technetium-99m-labeled *Escherichia coli* and 8-3H-adenine-labeled *Salmonella typhimurium* [142,143].

In conclusion, bacteria have distinctive advantageous properties compared to other cell-based targeting agents. Specifically, low production costs, a variety of suitable species, ease of manipulation and culturing, and selective replication in tumors together suggest great potential for bacterium-based imaging. However, since bacteria are microorganisms that could elicit destructive immune responses, vigilance should be maintained throughout their development, implementation, and clinical use.

### 3.3. Virus-Based IGS Carriers

Although not regarded as living, viruses are ideal candidates for tumor imaging considering their specificity, small diameter (5–400 nm), and ability to self-replicate. Oncolytic virus therapy, whereby cancer patients are deliberately infected with viruses that selectively kill malignant cells and spare heathy tissue, is a promising therapeutic approach [144]. The viruses used in this context are naturally occurring strains that are sometimes genetically modified. These viruses are specifically directed against cancer tissue and replicate within tumor cells to produce viral progeny. Thus, the virus itself acts as an active drug. A noteworthy example is the glioma-specific Herpes simplex type 1 amplicon virus (SU4-124 HSV-1) used by Delwar et al. [145]. They reported that the virus exhibited strong anti-tumor effects against glioma cell lines in vitro and, in mice bearing human glioma U87 tumors, after intratumoral injection. Importantly, no viral DNA was detected in healthy tissue.

A major concern that is generally associated with the use of viruses for therapy or diagnosis is that common, naturally occurring viruses that lead to viremia are likely to trigger the production of neutralizing antibodies in the patient via memory-induced immunity. However, this would not be an issue with viral agents used for (pre)operative targeted imaging in IGS, as the virus would be administered to the patient shortly before surgery for short-term use only (a few minutes to several hours). Namely, the patients’ anti-viral immune responses would require at least a couple of days to reach peak intensity. One could even argue that these immune responses favor the patient in terms of toxicity, since the mechanism will ensure removal of the virus from the circulation. Next to that, anti-viral agents, when available and effective against a specific viral strain, could be easily administered directly after the clinically needed effect, ensuring safety. Nevertheless, it should be kept in mind that these anti-viral agents are not available for and effective against all virus species (e.g., variable anti-viral activity against adenoviruses).

Adenoviruses or adeno-associated viruses that are used as vectors for gene therapy can be adopted as a delivery system for macromolecules other than DNA, including for the delivery of imaging agents to tumors, owing to the amenability of their capsids to surface modification [144]. For instance, Shan and co-authors functionalized adenovirus capsids with folate to enhance the viruses’ natural ability to target tumors that express low levels of Coxsackie-adenovirus receptor [146]. The authors attributed this effect to the increase in expression of folate receptors in cancer cells of malignant tumors during proliferation and cellular activation (e.g., cancers of the brain, lung, testis, kidney, colon, myelocytic blood cells, and liver) [146]. They also attached the NIRF dye ICG-Der-02 to their modified adenovirus, which allowed for in vivo fluorescence imaging. Furthermore, the authors hypothesized that it should be possible to extend the targeting capability of adenoviruses by other chemical modifications of its capsid [146]. Spronken et al. engineered five different reporter viruses of the influenza A virus, including one that was transfected with a near-infrared fluorescent protein [147]. Moreover, Hofherr et al. succeeded in the real-time tracking of intravenously injected adenoviruses with a near-infrared fluorophore in mice [148]. They preclinically administered the clinically used fluorophore IRDye800CW in a concentration of 10,000–100,000 dye molecules per viral particle. The fact that they performed the dye-coupling reaction at room temperature for only 1 hour suggests that their approach would be feasible in a clinical setting. In the context of SPECT-CT, it was shown by Stella Man et al. that systemically administered Iodine-125-labeled oncolytic adenoviral mutants could be monitored in real time using murine pancreatic cancer models [149]. The researchers designed a radiolabeling procedure with optimized conditions, retaining the adenoviral tumor-targeting functions.

Everything considered, the combination of their ease of modifiability, ability to replicate, specificity, and variety in suitable species render viruses as interesting candidates for IGS targeting agents. However, two important considerations should be made when choosing a specific virus species to focus on in further research: (i) there should be either an effective anti-viral agent available or an eliminated risk of adverse (immune) responses, and (ii) the individual patients’ immune reaction against the specific virus should be within reasonable margins, without prematurely neutralizing the virus’ imaging capabilities. In general, when considering current developments, the immunogenicity of viruses should not be a major concern for short-term imaging applications such as IGS.

## 4. Synthetic Nanoparticles for IGS

Synthetic nanoparticles such as liposomes, dendrimers, and carbon nanotubes have been designed for the targeting of tumors at multiple sites [150]. These particles are substantially larger (Figure 2 and Table 1) than molecular targeting agents such as peptides (≈1 nm) and IgG antibodies (10 nm) [151], and they are not as flexible as living cells, but they are still able to enter endothelial pores (fenestrae ≈10 nm–2 μm) and penetrate (tumor) tissues efficiently [152]. This phenomenon is partly attributed to the enhanced permeability and retention (EPR) effect, by which neoangiogenic vasculature favors the permeability and retention of extravascular molecules within the stroma. Nanoparticles can be engineered with a high level of control to perform physiological tasks such as the delivery of drugs or dyes to tumors [151]. The size of nanoparticles influences their distribution in tissue; thus, larger nanoparticles (50–200 nm) tend to have greater passive accumulation at tumor sites but less efficient penetration into the tumor, whereas smaller nanoparticles (<50 nm) penetrate easily into the tumor but are more rapidly eliminated from the circulation. Common disadvantages of nanoparticles include rapid clearance by macrophages and the liver (within three to five hours), a need for large numbers of nanoparticles for effective tumor labeling, high production costs, and limitations on the amount of targeting agents that can be added to the surface [92,153]. As synthetic objects, nanoparticles are neither living nor cell-derived. However, they can be coated with the membranes of cells of various origins. Therefore, we will include a short overview of the most relevant types of nanoparticles and their application for imaging purposes.

Liposomes are nanoparticles enclosed by a modifiable lipid-bilayer membrane, which enables them to carry both hydrophobic and hydrophilic cargo, potentially bypass the reticuloendothelial system (by which phagocytic cells remove immunogenic particles from the circulation), and undergo surface functionalization. Examples of liposomes used for targeted therapies include work by the group of Basel, who modified liposomes with peptides that specifically target metastases and reported the superior targeting and efficacy of metastases compared to highly metastatic primary tumors [154,155]. Interestingly, Li and Huang demonstrated that the uptake of liposomes by the reticuloendothelial system can be diminished and, consequently, accumulation of them in tumors increased by first adding a second bilayer to the liposomes and then functionalizing those with PEGylated lipids [156]. They also reported that this modification confers the liposomes with greater stability, minimal protein binding, and a neutral charge.

Dendrimers are nanoparticles comprising a central core and repeated branches with terminal functional groups. Their distinct tree-like shape, molecular weight range, and constitution provide them with unique features. For instance, dendrimers can carry drugs or dyes through encapsulation or via binding by electrostatic or hydrophobic/hydrogen bonds. Their main advantage is that they are assembled stepwise, enabling researchers to selectively introduce structural modifications for desired functionalities. The tumor cells can be targeted either by employing the EPR effect, by specific terminal functional groups (e.g., peptides or antibodies), or even a combination of the two [157]. An interesting example of employing dendrimers for tumor-targeting was reported by Sunoqrot et al., who encapsulated folate receptor-targeting polydendrimers into larger polyethylene glycol-poly lactic acid (PEG-PLA) polymeric nanoparticles [158]. The resultant dendrimer/nanoparticle hybrid platform executed controlled release of the folate receptor-targeting dendrimers in mice that bore folate receptor-overexpressing tumors. Importantly, the dendrimer–nanoparticle hybrid prevented premature renal elimination of the dendrimers in vivo, which is a problem that occurred when the free dendrimers had been administered. The authors expected that their folate receptor-targeting dendrimer–nanoparticle hybrid platform would exhibit superior targeting relative to that of basic polymeric nanoparticles.

Carbon nanotubes (CNTs) are hollow tubes of single or multilayered graphene whose structure confers them with high capacity for the delivery of bioactive agents and dyes. While the length and structure of CNTs depend on their design, both practical and theoretical studies have shown that the smallest possible carbon nanotube has a diameter of 3–4 Å [159,160,161]. The main downsides of CNTs are their high toxicity, poor solubility, and tendency to agglomerate, all of which limit their utility in clinical practice. A solution could be coating their exterior. CNTs are able to form covalent or non-covalent bonds with biological and bioactive species of chemical agents to become more soluble and less immunogenic. Their surface binding potential enables them to bind to targeting agents or other compounds of functional interest [162]. Wu and Zhao generated composites from single-walled CNTs (SWCNTs) and natural biopolymers for targeted imaging of cancer cells, showing that these composites provided an alternative to existing unbound dyes [163]. These nanoprobes (PPa/fluorescein isothiocyanate (FITC)-SWCNT-Folate), which target folate receptor-positive tumor cells, demonstrated good targeting and photodynamic therapy performance [163].

The field of nanoparticles for IGS is too rapidly expanding to be fully covered in this review, but its innovative nature is particularly illustrated by the following two examples. Vankayala et al. effectively imaged intraperitoneal ovarian tumors in mice using NIRF dye-labeled, virus mimicking nanoparticles, which were derived from genome-depleted mosaic viruses and were functionalized with anti-HER2 antibodies [164]. Schmidt et al. reviewed the characteristics of microrobots, which are motile microsystems that are designed to reach their targets after physical, biological, or chemical alterations [165]. Microrobots are highly engineerable and may consist of cell-made, synthetic, and hybrid components. This allows for a direct and active targeting potential via specific accumulation (e.g., mediated by active sensing of the (micro)environment) and increased penetration of healthy as well as diseased tissue.

## 5. Discussion and Overall Conclusions

Targeted imaging of pathologic lesions is an effective tool to enhance the visual capabilities of surgeons during operations. Cells, cell fragments, microorganisms, and nanoparticles, which have been labeled with NIRF dyes (e.g., ICG and IRDye800CW) MRI labels (e.g., magnetic beads), or radioactive labels (e.g., indium-111 oxine and 18F-FDG), might offer several advantages in comparison to currently used agents, as they can actively target lesion sites, penetrate target tissues, and interact with dynamic environments. Autologous cells, as well as platelets or other cell fragments, have the advantage that they are non-immunogenic, enabling them to avoid both unwanted immune responses and rapid removal from the circulation in treated patients. Although the use of any microorganism implies certain risks associated with immunogenicity, they also offer some specific advantages. For example, bacteria can be easily grown and selected/manipulated, and viruses are very small and readily modified, enabling them to target tumor sites with higher specificity than other agents. Platelets also excel at targeting both vascular and oncologic pathologies, which is mainly due to their size and natural tendency to bind to factors related to tumor tissue and damaged endothelial cells.

Regardless of the potential of any given new cellular or cell-derived tracer for IGS, a decisive factor for exploration is the feasibility of translating that potential into the clinic. In this sense, some of the presented modalities are facing issues in terms of balance between risk for the patient and benefit for IGS results. Research within the field of cell-based IGS has mainly focused on proof of principle preclinical experiments, while more translational research is needed to demonstrate the benefits of active targeting. Together with obvious disadvantages, such as the possibility of unwanted immune reactions by using bacteria or viruses and the immaturity and costs associated with production processes of autologous cell-based carriers, the adoption of this novel type of IGS tracer in the clinic will rely on both the support of clinicians and sufficient funding. In a later stage, another problem may arise in terms of applicability. Even when properly developed and affordable, the usage of different cell-based carriers for every specific combination of tumor type and patient characteristics will severely impair its ability to get adopted into the market. Regarding the latter, the different modification steps needed in some of the applications, such as purification or isolation, could add to its cumbersomeness and question its efficiency. Moreover, the risk of adverse immune reactions should not be considered lightly, since it will both add to the complexity of patient-specific treatment and accessory costs. In that respect, IGS will probably depend on the rapid developments in cell-based therapy.

Whilst one could argue that, with time, the general expenses of any specific technique decrease, the many factors that influence costs must be considered. Development processes could be drastically advanced, especially when other clinical research fields simultaneously drive the development of certain techniques. Therefore, the translation of new ISG tracers into the clinic will require that they are embraced, promoted, and co-developed by clinicians. The establishment of multidisciplinary research groups that bridge the implementation gap by drawing on the preclinical and clinical expertise of diverse professionals is essential. Furthermore, together with the development of novel implementations, early adoption and clinical use will increase demand, thereby facilitating acquisition of sufficient funding for the required experiments to regenerate the development cycle.

Currently, tracers for IGS are based on the targeting of a single protein by a labeled peptide or antibody. In the future, an ideal situation for new targeted tracers in IGS would be one of choice, in which the appropriate agent would be selected and modified based on the type of cancer or vascular lesion, also taking into account its anatomic location, any relevant comorbidities, and patient preferences. To achieve this, the development and evaluation of multiple types of cell-based targeting agents should help ensure success for patients, clinicians, and researchers, as such agents can inform each other and might even be used concomitantly for increased accuracy. While the risk–benefit ratio of cell-based IGS still needs to be carefully weighed, the field is still in its infancy and has enormous potential. There are many other possibilities to develop new tracers for IGS beyond those that we have reviewed here. A non-obvious but fascinating choice for targeted imaging are the algae-based systems as recently developed by Qiao and coworkers [166]. Therefore, and referring to other famous explorations, we regard image-guided surgery as one of the most exciting and clinically translational fields in future medicine and are proud to participate in “a mission to explore novel targets and develop new tracers, and to boldly go where no man (or tracer) has gone before [167].”

## Figures and Tables

**Table 1:** Main characteristics, advantages, and disadvantages of cell- and nanoparticle-based tracers for image-guided surgery.

	Macrophages/ Monocytes	Neutrophils	(CAR-)T Cells	(CAR-)NK Cells	Platelets	MSCs	Exosomes	Bacteria	Viruses	Nanoparticles
*Diameter*	16–22 μm	9–15 μm	6–12 μm	6–7 μm	1–3 μm	15–30 μm	40–100 nm	0.5–10 μm	5–400 nm	20–200 nm
*Biocompatibility*	+ + +	+ + +	+ +(+)	+ + +	+ + +	+ + +	+ + (+)	- - -	- -	+/−
*Targeting potential*	+ +	+ +	+ + +	+ + +	+	+	+	+ +	+ + +	+ + +
*Feasibility &* *applicability*	+	+/−	-	+/−	+	+/−	+/−	+/−	+	+
*Specific advantages*	1. Active tumor-homing 2. Easily labeled	1. Active tumor-homing 2. Abundance in blood	1. CAR specificity	1. CAR specificity 2. Less associated with off-target effects (compared to T cells) 3. Relatively inexpensive (compared to T cells)	1. Penetrative capabilities 2. Availability in the circulation	1. Fast ex-vivo expansion 2. Relatively easy acquisition	1. Non-immunogenic 2. Penetrative abilities 3. Physiochemical stability	1. Easily genetically manipulated and cultured 2. Low production costs 3. Tumor-specific replication 4. Variety in suitable species	1. Easily modified 2. Variety in suitable strains 3. Tumor specificity 4. Ability to replicate	1. Multitude of design possibilities 2. High specificity potential 3. Humanization to increase biocompatibility 4. High surface binding potential
*Specific challenges*	1. Avoiding disease promoting subtypes 2. Modification process-induced cell alterations	1. Acquisition 2. Fragile & easily activated in culture 3. Modification process-induced cell alterations	1. Costs of cell acquisition and modification 2. Efficiency of cell acquisition and modification 3. Harmful off-target responses 4. Modification process-induced cell alterations	1. Relative inefficiency of gene transduction methods 2. Relative lack of NK cell-specific CARs 3. Modification process-induced cell alterations	1. Platelet aggregation 2. Platelet-tumor cell interactions	1. Trapping in lungs, liver, and spleen 2. Coagulation inducing properties 3. Modification process-induced cell alterations	1.Purification techniques; yield 2. Costs of large scale production	1. Patho- and immunogenecity	1. Memory-induced immune responses (neutralizing antibodies) 2. Patho- and immunogenicity 3. Species-specific non-availability of anti-viral agents	1. Large pools needed 2. Rapid clearance 3. Expensive manufacturing and processing 4. Limited amount surface targeting agents

-, +/−, +, ++, +++ indicate increasing compatibility, potential, or applicability.

## Data Availability

Not applicable.

## Abbreviations

ApoE	Apolipoprotein E
B cell	Bursa of Fabricius cell
BCG	bacillus Calmette–Guérin
CAR	Chimeric antigen receptor
CCL2	C–C motif chemokine ligand 2
CNT	Carbon nanotube
COPD	Chronic obstructive pulmonary disease
CT	Computed tomography
CTL	Cytotoxic T lymphocyte
Cy5	Cyanine 5
(im)DC	(immature murine) Dendritic cell
DiR	1:1′-dioctadecyl-3,3,3′,3′-tetramethylindotricarbocyanine iodide
DNA	Deoxyribonucleic acid
EGF(R)	Epidermal growth factor (receptor)
EPR effect	Enhanced permeability and retention effect
EV	Extracellular vesicle
FDG	Fluorodeoxyglucose
FITC	Fluorescein isothiocyanate
HER(2/neu)	Human epidermal growth factor receptor (2/neu)
HIV	Human immunodeficiency virus
HPF	Heparin, protamine, and ferumoxytol
HSV	Herpes simplex virus
ICG	Indocyanine green
IGS	Image-guided surgery
LAMP2b	lysosome-associated membrane glycoprotein 2b
MCP-1	Monocyte chemoattractant protein-1
MHC	Major histocompatibility complex
MIP	Macrophage inflammatory protein
MRI	Magnetic resonance imaging
MSC	Mesenchymal stromal cell
MV	Microvesicle
NIRF	Near-infrared fluorescence
NK cell	Natural killer cell
PEG-PLA	Polyethylene glycol-Poly lactic acid
PET	Positron emission tomography
RNA	Ribonucleic acid; mi = micro, si = short interfering, lnc = long non-coding
SPECT	Single photon emission computed tomography
SWCNT	Single-walled carbon nanotube
T cell	Thymus cell
Th	T helper
TNFα	Tumor necrosis factor α
VCAM-1	Vascular cell adhesion molecule-1
VEGF(R)	Vascular endothelial growth factor (receptor)
